# Effect of velocity loss squat induced post-activation performance enhancement on lower limb explosive power in sprinters

**DOI:** 10.3389/fphys.2026.1725012

**Published:** 2026-02-25

**Authors:** Jiawei Sun, Lin Deng, Shiyi Xu, Jianing Gu, Jiayi Li, Ruofei Wang, Xinyu Lu, Nan Lou, Jianghua Zou, Zhanming Xu, Laikang Yu

**Affiliations:** 1 College of Education, Beijing Sport University, Beijing, China; 2 Tongliao No.5 Senior High School, Tongliao, China; 3 College of Physical Education, Huaqiao University, Quanzhou, China; 4 Key Laboratory of Sport Training of General Administration of Sport of China, General Administration of Sport, Beijing, China; 5 School of Competitive Sports, Beijing Sport University, Beijing, China; 6 Beijing Key Laboratory of Sports Performance and Skill Assessment, Beijing Sport University, Beijing, China; 7 Department of Strength and Conditioning Assessment and Monitoring, Beijing Sport University, Beijing, China

**Keywords:** countermovement jump, lower limb explosive power, post-activation performance enhancement, post-activation potentiation, sprinter, velocity loss

## Abstract

**Background:**

This study aimed to identify the optimal velocity loss (VL) threshold during squats for inducing post-activation performance enhancement (PAPE) in track and field sprinters, with the goal of maximizing sprint performance.

**Methods:**

Twenty-four sprinters performed squat-based PAPE protocols using 85% 1RM (1 Repetition Maximum) across four VL thresholds (5%, 10%, 15%, and 20%). The 30-m sprint and countermovement jump (CMJ) tests were administered at baseline and at 4, 8, 12, and 16 min post-intervention. Measurements included CMJ height, peak power, momentum, and the number of squats completed under each VL condition.

**Results:**

The 5% VL condition led to significant improvements in 30-m sprint time at 4 min (F_(1,47)_ = 7.292, P = 0.01, Cohen’s d = −0.777) and 8 min (F_(1,47)_ = 4.603, P = 0.037, Cohen’s d = −0.615), along with increases in CMJ height (F_(1,47)_ = 5.748, P = 0.021, Cohen’s d = 0.69), peak power (F_(1,47)_ = 5.585, P = 0.022, Cohen’s d = 0.685), and momentum (F_(1,47)_ = 6.462, P = 0.014, Cohen’s d = 0.715). Under the 10% VL condition, significant gains were observed in 4-min sprint performance (F_(1,47)_ = 5.288, P = 0.026, Cohen’s d = −0.656) and CMJ peak power at 4 min (F_(1,47)_ = 5.585, P = 0.014, Cohen’s d = 0.741) and 8 min (F_(1,47)_ = 3.884, P = 0.022, Cohen’s d = 0.687). The number of squats performed was significantly lower at 5% VL compared to other thresholds (P < 0.001).

**Conclusion:**

A velocity loss threshold of 5% during squats at 85% 1RM elicits a significant PAPE effect by 4 min post-exercise, with the fewest required repetitions. For practical application, a recovery interval of 4–8 min is recommended to optimize training outcomes in sprinters.

## 1 Introduction

Sprinting is one of the most competitively distinctive events in track and field, requiring exceptionally high levels of lower-limb explosive power. Within this context, both speed and strength are critical determinants of performance, and even marginal improvements in explosive capacity can substantially influence competitive outcomes.

Post-activation Potentiation (PAP) has been recognized as a unique physiological phenomenon of considerable relevance to sports performance enhancement. PAP refers to a transient augmentation of muscle force, contraction velocity, and explosive power following maximal or near-maximal exercise, primarily mediated by the phosphorylation of myosin regulatory light chains in type II muscle fibers (Brown and Loeb, 1998). A substantial body of evidence indicates that PAP can yield both immediate and lasting benefits, whether incorporated into warm-up routines or contrast training programs (Bishop, 2003; DeRenne, 2010; Maio Alves et al., 2010; García-Pinillos et al., 2014; Smith et al., 2018; Pagaduan et al., 2019).

Closely related but mechanistically distinct is post-activation performance enhancement (PAPE), defined as the acute improvement in strength, speed, or power output following a single bout of high-intensity exercise (Blazevich and Babault, 2019; Zimmermann et al., 2020). While PAP is primarily induced by electrical stimulation and enhanced actin–myosin cross-bridge efficiency, PAPE is associated with physiological factors such as increased muscle temperature, intracellular water content, and motor unit recruitment. Accordingly, the onset and magnitude of PAP and PAPE differ (Hodgson et al., 2005; Wilson et al., 2013). Importantly, PAP does not invariably result in PAPE; the latter may arise from PAP or occur independently, reflecting their distinct underlying mechanisms (Ah Sue et al., 2016; Dobbs et al., 2019). Fatigue can be readily attained during PAPE-inducing protocols and may accumulate primarily in the specific muscle groups involved in the exercise, resulting in a localized effect. This suggests that the potential interference of such local fatigue on the performance of the target muscles must be fully considered when evaluating the net potentiation effect (Stastny et al., 2024).

Strength training not only plays a fundamental role in improving athletic performance and reducing injury risk (McGuigan et al., 2012; Suchomel et al., 2016), but also contributes to health promotion in recreational exercisers and in the management of chronic diseases (Kamada et al., 2017; Piercy and Troiano, 2018). Furthermore, it is a central method for inducing PAPE. The magnitude of training benefits depends heavily on the precise regulation of load intensity and volume (Kraemer and Ratamess, 2004). However, traditional approaches, such as prescribing loads based on a percentage of one-repetition maximum (%1RM) or using maximum repetitions (RM), have limitations. Maximum strength testing (%1RM) can be impractical, time-consuming, and associated with injury risk (González-Badillo et al., 2017), whereas training to failure (RM) may hinder improvements in explosive power and impair rate of force development (Andersen and Aagaard, 2000; Izquierdo et al., 2006).

In response to these limitations, velocity-based training (VBT) has emerged as a promising method for quantifying and regulating training load (González-Badillo and Sánchez-Medina, 2010; González-Badillo et al., 2011). Originally proposed by González-Badillo in 2010, VBT enables real-time monitoring of movement velocity to optimize strength training prescription and manage fatigue (González-Badillo and Sánchez-Medina, 2010; Mann et al., 2015). Previous research has showed that greater velocity loss (VL) during a set is associated with higher ratings of perceived exertion (RPE), increased blood lactate accumulation, and decreased post-exercise jump performance (Weakley et al., 2020). Importantly, VBT facilitates adequate training stimuli while minimizing excessive fatigue (González-Badillo and Sánchez-Medina, 2010).

Recent meta-analyses further suggest that the magnitude of VL plays a decisive role in training outcomes. Specifically, low-to-moderate VL thresholds (<25%) are associated with performance maintenance and reduced fatigue, whereas higher VL thresholds (>25%) appear to favor hypertrophic adaptations (Hernández-Belmonte and Pallarés, 2022; Hickmott et al., 2022). Nevertheless, whether VBT can be effectively applied to PAPE in the explosive power of sprinters remains unclear. Traditional approaches relying on %1RM or fixed repetition schemes often fail to account for individual variations in fatigue, potentially compromising the induction of PAPE (Yuan et al., 2023). By contrast, monitoring VL provides a more individualized means of regulating training volume, optimizing the potentiation stimulus, and reducing injury risk.

A substantial body of research confirms that targeted conditioning protocols, such as high-intensity back squats, can effectively enhance performance in explosive movements like jumping and sprinting (Brink et al., 2022; Loturco et al., 2024). However, a consensus on the optimal protocol remains elusive. While some studies advocate for moderate-intensity, multi-set schemes (Chen et al., 2023), others emphasize the potential superiority of low-volume, maximal intensity stimuli (Maroto-Izquierdo et al., 2020). This disagreement stems from the critical role of fatigue accumulation, which is widely recognized as a key factor that can undermine or even mask the PAPE effect. Consequently, the core of successfully inducing PAPE lies in precise fatigue management. Although VBT has demonstrated its precision in load regulation via velocity monitoring within the strength training domain, its application value and specific efficacy in regulating PAPE inducing protocols for sprinters have not been sufficiently investigated or clearly established.

Accordingly, the present study aims to investigate the effects of four low-to-moderate VL thresholds (5%, 10%, 15%, 20%) on lower-limb explosive performance in sprinters. We hypothesize that a 5% VL condition will elicit superior improvements in jump and sprint performance compared with higher VL thresholds (10%–20%).

## 2 Materials and methods

### 2.1 Participants

The required sample size was estimated using G*Power software (version 3.1.9.7; Franz Faul, University of Kiel, Kiel, Germany) repeated-measures ANOVA, d = 0.25, α = 0.05, power = 0.80 (Han et al., 2025), indicating a minimum of 20 participants. With reference to McKay’s training level classification (McKay et al., 2022), athletes classified as Tier 2 were included. A total of 24 male sprinters voluntarily enrolled in the study during the off-season (Table 1). During this period, the athletes were not in complete rest but followed a structured maintenance training program consisting of 3–4 sessions per week. The program to ensure baseline fitness stability and avoid acute fatigue from high intensity training or competition. Written informed consent was obtained from all participants after fully informed of the study purpose, procedures, potential risks, and possible discomforts. Prior to data collection, participants were familiarized with all testing protocols. Participants were free to withdraw from the study at any time. No participants had a history of cardiovascular or respiratory diseases, nor any lower limb joint injuries in the 6 months preceding the study, and all were in good health at the time of testing. All participants had at least 3 years of prior experience in both squatting and sprint training. To minimize consuming factors, they were instructed to abstain from alcohol and caffeine intake, avoid high-intensity resistance exercise, and ensure adequate rest within 24 h before testing and ensured at least 8 h of sleep. The study was approved by the Ethics Committee of Beijing Sport University (Approval No.: 2025266H) and conducted in accordance with the Declaration of Helsinki.

**TABLE 1:** Physical characteristics of subjects at baseline (n = 24).

Variables	Mean ± SD
Age (years)	20 ± 1.05
Height (cm)	180 ± 4.56
Body mass (kg)	71 ± 5.02
Resistance training experience (years)	4.5 ± 1.59
1RM (kg)	130 ± 13.59
1RM/Body mass	1.83 ± 0.15

RM: repetition maximum.

### 2.2 Study design

A repeated-measures, randomized crossover design was employed to evaluate the effects of squat-induced PAPE on CMJ and 30-m sprint performance. The allocation of different variables was achieved using randomization software (Research Randomizer, www.randomizer.org) and carried out by a researcher not involved in data collection. Four low-to-moderate VL thresholds (5%, 10%, 15%, and 20%) were tested. After completing a 1RM squat test, participants underwent all experimental sessions at the same time of day, with at least 48 h between sessions to minimize fatigue. Each participant completed the PAPE squat protocol under all thirty-two experimental conditions (4 VL thresholds × 4 post-activation time points × 2 dependent measures). For each condition, CMJ and 30-m sprint tests were performed both before and after the conditioning activity to evaluate the PAPE effect.

### 2.3 Procedures

#### 2.3.1 1RM squat test

In the first testing session, the participants’ height and body weight were initially measured. Subsequently, all participants completed a familiarization process with the VBT testing protocol to ensure proper use of the velocity monitoring equipment and adherence to the testing movement requirements. Following this, the free weight barbell back squat 1RM test was conducted. After a standardized warm-up (Table 2), testing commenced with an initial load of 40 kg. When mean velocity (MV) dropped below 0.5 m/s, the load was progressively increased in 20 kg increments. Squat technique strictly adhered to National Strength Conditioning Association (NSCA) guidelines (Yuan et al., 2023): feet shoulder-width apart, barbell placed on the upper trapezius, neutral lumbar spine, knees aligned with toes during descent until thighs reached parallel, and controlled ascent with trunk stability. MV approached 0.5 m/s, incremental load increases of 1–5 kg were used to precisely determine the 1RM.

**TABLE 2:** Warm-up programs for PAPE protocol.

Warm-up component	5, 10, 15, 20% VL
Jogging	5 min
Muscle activation	Shoulder circles: 1 set × 12 reps Mountain climber: 1 set × 18 reps Single leg glute bridges: 1 set × 12 reps Superman: 1 set × 8 reps Squat: 1 set × 8 reps
Dynamic stretching	Inchworm: 1 set × 8 reps 90/90 for hips: 1 set × 8 reps Lunge walk: 1 set × 8 reps Walking knee lift: 1 set × 8 reps
Neuromuscular activation	Split squat jump: 1 set × 5 reps Squat jump: 1 set × 5 reps Box jump: 1 set × 5 reps

The maximum load lifted under strict adherence to proper technique was recorded as the 1RM. For each attempt, participants were instructed to perform the eccentric phase in a self-controlled manner and to execute the concentric phase with maximal intent for rapid force production. Certified spotters ensured safety throughout. Based on MV, attempts were categorized as light (MV > 0.7 m/s), moderate (0.5 m/s ≤ MV ≤ 0.7 m/s), or heavy (MV < 0.5 m/s), with 3, 2, and 1 attempt permitted, respectively. Rest intervals were 4 min for light and moderate attempts and 6 min for heavy attempts (Yuan et al., 2023).

#### 2.3.2 PAPE test

**FIGURE 1:**
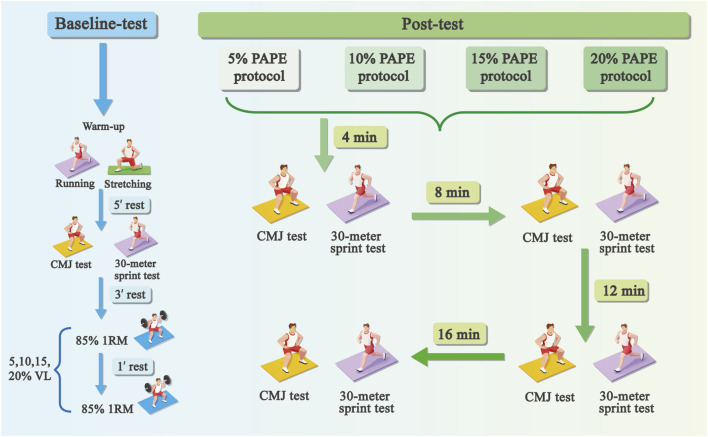
Schematic representation of PAPE test. Note: PAPE, post-activation performance enhancement; CMJ, countermovement jump.

As illustrated in Figure 1, participants completed the eight experimental sessions in a randomized balanced order. Each session began with a standardized warm-up, followed by a 5-min rest. Participants then performed three CMJ trials and one 30-m sprint (best CMJ recorded as baseline). After a 3-min rest, the PAPE intervention was performed, consisting of two sets of squats at 85% 1RM (Seitz and Haff, 2016), consisting of a 1-min rest interval (Saez Saez De Villarreal et al., 2007; Tsolakis et al., 2011). A 5 cm marker line was placed 30 cm behind the barbell, with participants aligning their toes on the line. Feet were positioned shoulder-width or slightly wider, with toes slightly outward. A 1-m-long elastic band was suspended 50–70 cm above the line and adjusted to the height corresponding to parallel thigh position.

Squat velocity was continuously monitored using a GymAware Power tool linear position transducer (Kinetic Performance, Australia). The device was placed to the right of the squat rack, collecting data via a vertical cable. Verbal encouragement and real-time velocity feedback were provided during each repetition. The eccentric phase of the squat was controlled at a moderate tempo, with a target velocity range of 0.5–0.7 m/s (Stastny et al., 2024). The velocity loss threshold was calculated as: [Terminal velocity = Initial velocity × (1–VL%)], (Note: Initial velocity refers to the first repetition of each set.) (González-Badillo et al., 2017). Each set was terminated once repetition velocity fell below predetermined threshold (Sindiani et al., 2020). The protocol was based on evidence indicating that high-intensity loads (≥85% 1RM) and multiple sets maximize PAPE, and that a 1-min inter-set interval enhances potentiation (Saez Saez De Villarreal et al., 2007; Tsolakis et al., 2011). Following the squat intervention, CMJ and 30-m sprint tests were administered at 4, 8, 12, and 16 min post-intervention.

#### 2.3.3 Countermovement jump (CMJ)

At each post-intervention time point, participants performed three CMJ trials with 10-s rest intervals. Participants stood upright on a force platform with their hands on hips and feet shoulder-width apart. On the “jump” command, they descended to a self-selected depth before executing a maximal vertical jump, maintaining straight legs and hands on hips during flight (Hernández-Belmonte and Pallarés, 2022). The best trial was recorded for analysis. A three-dimensional force platform (Kistler 9281CA, Switzerland, 1,000 Hz) collected jump variables including height, peak power, and takeoff momentum (McMahon et al., 2020). Verbal encouragement was provided, and environmental conditions were maintained at 20 °C–24 °C (Yuan et al., 2023).

#### 2.3.4 30-m sprint test

One 30-m sprint was performed at baseline and at each of the four post-intervention time points. Participants assumed a standardized starting posture: feet evenly spaced, the front foot 50 cm behind the start line, arms relaxed at sides, and hips and knees slightly flexed. To minimize reaction-time influence, sprints were self-initiated. Participants were instructed to exert maximal effort throughout. Verbal encouragement was provided throughout the testing period. Timing gates (SmartSpeed Dash, PB1281, Australia) were positioned at the start and finish lines, 1 m above ground, and times were recorded to the nearest 0.01 s (Xie et al., 2022; Liu et al., 2024). Verbal encouragement was provided, and environmental conditions were maintained at 20 °C–24 °C (Yuan et al., 2023).

### 2.4 Statistical analysis

Descriptive data are presented as mean ± standard deviation (Mean ± SD). Reliability of CMJ and sprint measures was assessed using the coefficient of variation (CV) and intraclass correlation coefficient (ICC) (Cormack et al., 2008). Reliability thresholds were defined as CV <15% and ICC interpreted as poor (<0.50), moderate (0.50–0.74), good (0.75–0.90), or excellent (>0.90) (Koo and Li, 2016). A repeated-measures ANOVA was conducted to evaluate CMJ and sprint performance. Where significant main or interaction effects were observed, Newman-Keuls *post hoc* tests were applied. Significance was set at p < 0.05. Effect sizes (Cohen’s d) were calculated (Koo and Li, 2016), with magnitudes classified as trivial (0–0.2), small (0.2–0.6), moderate (0.6–1.2), and large (>1.20). Statistical analyses were performed using SPSS version 23.0 (IBM Corp, United States), and figures were generated with GraphPad Prism version 8.3.0 (GraphPad Software, United States).

## 3 Results

### 3.1 Jump momentum, jump height, and peak power output

**FIGURE 2:**
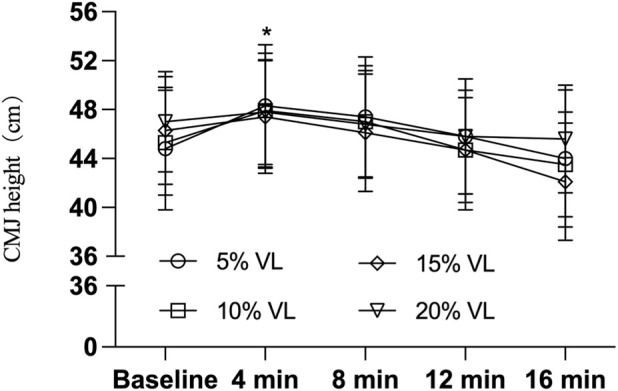
CMJ height during PAPE condition of different VL (n = 24). Note: * indicates P < 0.05.

**FIGURE 3:**
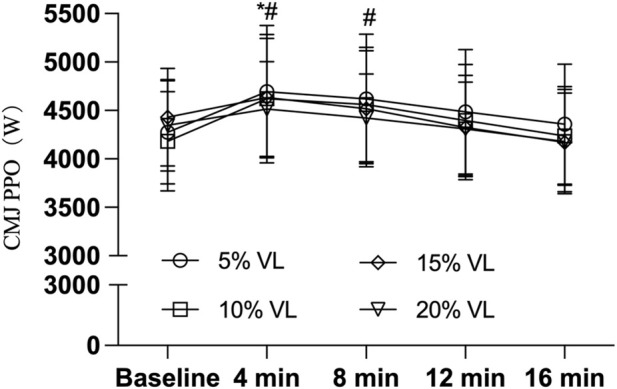
CMJ PPO during PAPE condition of different VL (n = 24). Note: * indicates P < 0.05, ^#^ indicates P < 0.05.

**FIGURE 4:**
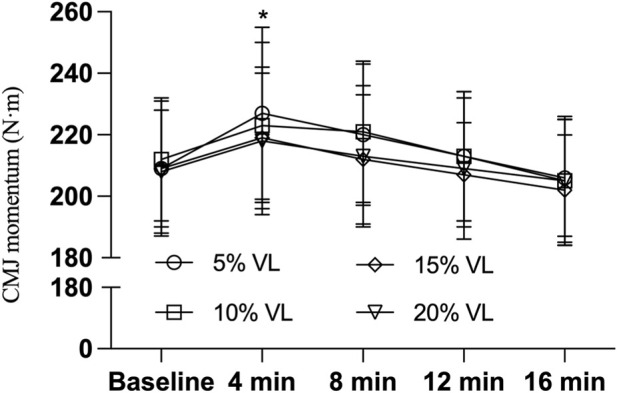
CMJ momentum during PAPE condition of different VL (n = 24). Note: * indicates P < 0.05.

As shown in Table 3, the 5% VL condition elicited significant improvements in CMJ height (F_(1,47)_ = 5.748, P = 0.021, Cohen’s d = 0.690), PPO (F_(1,47)_ = 5.585, P = 0.022, Cohen’s d = 0.685), and momentum (F_(1,47)_ = 6.462, P = 0.014, Cohen’s d = 0.715) at the 4-min post-intervention time point. Additionally, the 10% VL condition also produced significant enhancements in CMJ PPO at both 4 min (F _(1,47)_ = 5.585, P = 0.014, Cohen’s d = 0.741) and 8 min (F _(1,47)_ = 3.884, P = 0.022, Cohen’s d = 0.687) post-intervention. However, no significant effects were observed for CMJ height at 4 min (F_(1,47)_ = 3.826, P = 0.057, Cohen’s d = 0.563) or 8 min (F_(1,47)_ = 1.668, P = 0.203, Cohen’s d = 0.373), nor for momentum at 4 min (F_(1,47)_ = 2.244, P = 0.141, Cohen’s d = 0.463) or 8 min (F_(1,47)_ = 1.851, P = 0.180, Cohen’s d = 0.418) (see Tables 4, 5; Figures 2–4).

**TABLE 3:** 30-m sprint, Jump height, PPO and momentum of CMJ during PAPE condition of different VL (n = 24).

Sports performance variables	VL (%)	Assessment time point				
		Baseline	4 min	8 min	12 min	16 min
30 m (s)	5	4.09 ± 0.111	3.994 ± 0.135^a^	4.014 ± 0.135^a^	4.052 ± 0.132	4.071 ± 0.129
	10	4.031 ± 0.080	3.969 ± 0.107^a^	3.995 ± 0.108	4.04 ± 0.113	4.084 ± 0.104
	15	4.037 ± 0.094	3.979 ± 0.123	4.003 ± 0.121	4.034 ± 0.129	4.056 ± 0.112
	20	4.059 ± 0.085	4.015 ± 0.106	4.022 ± 0.11	4.056 ± 0.117	4.096 ± 0.118
CMJ height (cm)	5	44.78 ± 5.2	48.3 ± 5^a^	47.35 ± 4.91	45.81 ± 4.71	43.96 ± 5.61
	10	45.32 ± 4.29	47.85 ± 4.69	46.97 ± 4.55	44.68 ± 4.26	43.45 ± 4.26
	15	46.28 ± 4.43	47.39 ± 4.56	46.11 ± 4.8	44.65 ± 4.91	42.13 ± 4.79
	20	47.04 ± 4.12	47.81 ± 4.31	46.83 ± 4.4	45.8 ± 4.65	45.58 ± 4.41
CMJ PPO (W)	5	4,274 ± 532	4,693 ± 685^a^	4,618 ± 669	4,487 ± 643	4,359 ± 620
	10	4,182 ± 512	4,621 ± 663^a^	4,562 ± 591^a^	4,397 ± 580	4,238 ± 511
	15	4,431 ± 505	4,632 ± 612	4,517 ± 599	4,325 ± 538	4,171 ± 511
	20	4,348 ± 473	4,514 ± 490	4,422 ± 454	4,310 ± 484	4,179 ± 537
CMJ momentum (N·m)	5	209 ± 22	227 ± 28^a^	220 ± 23	213 ± 21	206 ± 19
	10	212 ± 20	223 ± 27	221 ± 23	213 ± 21	205 ± 21
	15	209 ± 19	219 ± 21	212 ± 21	207 ± 17	202 ± 18
	20	208 ± 20	218 ± 24	213 ± 23	209 ± 23	205 ± 20

^a^ indicates P < 0.05, showing a significant difference compared to the baseline value. PPO, peak power output; PAPE, post-activation performance enhancement; CMJ, countermovement jump.

**TABLE 4:** Descriptive statistics of the acute effects of different variables before and after four velocity loss interventions (n = 24).

Sports performance variables	VL (%)	Cohen’s d with 95%CI			
		4 min	8 min	12 min	16 min
30 m (s)	5	−0.777 (−1.364, −0.19)	−0.615 (-1.194, −0.036)	−0.312 (−0.881, −0.258)	−0.158 (−0.725, 0.409)
	10	−0.656 (−1.237, −0.075)	−0.379 (−0.95, 0.129)	0.092 (−0.474, 0.658)	0.571 (−0.006, 1.148)
	15	−0.53 (−1.105, 0.046)	−0.314 (−0.883, 0.255)	−0.027 (−0.592, 0.539)	0.184 (−0.383, 0.751)
	20	−0.458 (−1.031, 0.115)	−0.376 (−0.947, 0.194)	−0.029 (−0.595, 0.536)	0.36 (-0.211, 0.93)
CMJ height (cm)	5	0.69 (0.108, 1.272)	0.508 (−0.067, 1.083)	0.208 (−0.36, 0.775)	−0.152 (−0.718, 0.415)
	10	0.563 (−0.014, 1.14)	0.373 (−0.198, 0.944)	−0.15 (−0.716, 0.417)	−0.439 (−1.01, 0.135)
	15	0.247 (−0.321, 0.815)	−0.037 (−0.603, 0.529)	−0.349 (−0.919, 0.222)	−0.9 (−1.493, −0.306)
	20	0.183 (−0.384, 0.75)	−0.048 (−0.615, 0.517)	−0.282 (−0.851, 0.286)	−0.342 (−0.912, 0.228)
CMJ PPO (W)	5	0.685 (0.101, 1.265)	0.569 (−0.008, 1.146)	0.361 (−0.209, 0.931)	0.147 (−0.419, 0.714)
	10	0.741 (0.156, 1.326)	0.687 (0.105, 1.27)	0.393 (−0.178, 0.964)	0.109 (−0.547, 0.676)
	15	0.358 (−0.212, 0.929)	0.155 (−0.411, 0.722)	−0.203 (−0.77, 0.364)	−0.512 (−1.087, 0.063)
	20	0.345 (−0.225, 0.915)	0.16 (−0.407, 0.726)	−0.079 (−0.645, 0.487)	−0.334 (−0.904, 0.236)
CMJ momentum (N·m)	5	0.715 (0.131, 1.298)	0.489 (−0.085, 1.063)	0.186 (−0.318, 0.753)	−0.117 (−0.683, 0.45)
	10	0.463 (−0.11, 1.036)	0.418 (−0.154, 0.99)	0.049 (−0.517, 0.615)	−0.341 (−0.911, 0.229)
	15	0.499 (−0.075, 1.074)	0.15 (−0.417, 0.716)	−0.111 (−0.677, 0.455)	−0.378 (−0.949, 0.193)
	20	0.453 (−0.12, 1.026)	0.232 (−0.336, 0.8)	0.046 (−0.519, 0.612)	−0.15 (−0.717, 0.417)

PPO, peak power output; PAPE, post-activation performance enhancement; CMJ, countermovement jump.

**TABLE 5:** Reliability statistics (coefficient of variation and intraclass correlation coefficient) of sports performance indicators following different velocity loss interventions (n = 24).

Sports performance variables	VL (%)	CV (%) with ICC					
		Baseline CV (%)	4 min CV (%)	8 min CV (%)	12 min CV (%)	16 min CV (%)	ICC
30 m (s)	5	2.71	3.38	3.36	3.26	3.17	0.971
	10	1.98	2.70	2.70	2.80	2.55	0.942
	15	2.33	3.09	3.02	3.20	2.76	0.969
	20	2.09	2.64	2.73	2.88	2.88	0.961
CMJ height (cm)	5	11.61	10.35	10.37	10.28	12.76	0.939
	10	9.47	9.80	9.69	9.53	9.80	0.984
	15	9.57	9.62	10.41	11.00	11.37	0.978
	20	8.76	9.01	9.40	10.15	9.68	0.991
CMJ PPO (W)	5	12.45	14.60	14.49	14.33	14.22	0.990
	10	12.24	14.35	12.95	13.20	12.06	0.987
	15	11.40	13.21	13.26	12.44	12.25	0.993
	20	10.88	10.86	10.27	11.23	12.85	0.985
CMJ momentum (N·m)	5	10.53	12.33	10.45	9.86	9.22	0.985
	10	9.43	12.11	10.41	9.86	10.24	0.976
	15	9.09	9.59	9.91	8.21	8.91	0.980
	20	9.62	11.01	10.80	11.00	9.76	0.990

### 3.2 30-Meter sprint

**FIGURE 5:**
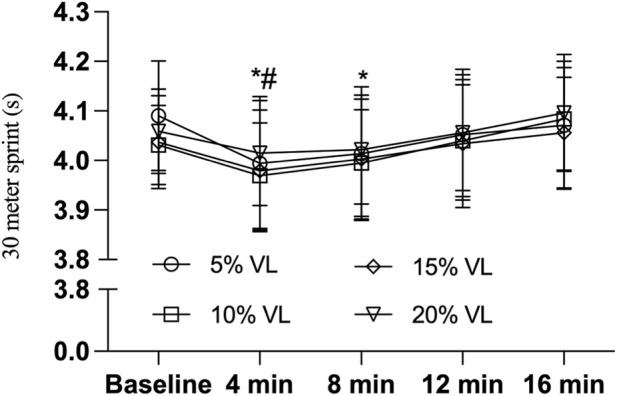
30-m sprint during PAPE condition of different VL (n = 24). Note: * indicates P < 0.05, ^#^ indicates P < 0.05.

As presented in Table 4, the 5% VL condition induced significant improvements in 30-m sprint performance at both 4 min (F _(1,47)_ = 7.292, P = 0.010, Cohen’s d = −0.777) and 8 min (F _(1,47)_ = 4.603, P = 0.037, Cohen’s d = −0.615) post-intervention. Similarly, the 10% VL condition yielded a significant enhancement at the 4-min time point (F _(1,47)_ = 5.288, P = 0.026, Cohen’s d = −0.656) (see Tables 5, 6; Figure 5).

### 3.3 Number of squat repetitions

As shown in Table 6, A one-way repeated-measures ANOVA revealed a highly significant main effect between groups in Set 1 (F _(3,47)_ = 39.196, P < 0.001). Post hoc tests indicated that the number of squat repetitions in the 10% VL group was not significantly different from that in the 5% VL group (P = 0.079), whereas the 15% VL and 20% VL groups showed highly significant differences compared to the 5% VL group (P < 0.001). In Set 2, there was also a highly significant main effect between groups (F _(3,47)_ = 34.913, P < 0.001). Post hoc comparisons showed a significant difference in squat repetitions between the 10% VL and 5% VL groups (P = 0.012), while the 15% VL and 20% VL groups exhibited highly significant differences compared to the 5% VL group (P < 0.001). For the total of both sets, a highly significant main effect between groups was observed (F _(3,47)_ = 71.328, P < 0.001). Post hoc analysis demonstrated a significant difference between the 10% VL and 5% VL groups (P = 0.004), with the 15% VL and 20% VL groups again showing highly significant differences compared to the 5% VL group (P < 0.001).

**TABLE 6:** The total number of repetitions in the first and second group of 4 VL conditions (n = 24).

VL (%)	Number of repetitions		
	Set 1	Set 2	2 sets in total
5	2.51 ± 0.58	2.75 ± 0.55	5.26 ± 0.82
10	2.97 ± 0.56^a1^	3.08 ± 0.67^a^	6.06 ± 1.01^aa^
15	4.10 ± 0.77^aaabbb^	4.33 ± 0.63^aaabbb^	8.43 ± 1.11^aaabbb^
20	5.39 ± 0.97^aaabbbccc^	5.23 ± 0.94^aaabbbccc^	10.63 ± 1.64^aaabbbccc^

In statistical notation, the symbol a denotes (p < 0.05), aa denotes (p < 0.01), and aaa denotes (p < 0.001). Similarly, bbb represents (p < 0.001); ccc represents (p < 0.001). The symbol a^1^ is used to indicate no statistically significant difference (p ≧ 0.05).

## 4 Discussion

This study aimed to investigate the effects of squat exercises performed with four distinct low-to-moderate VL thresholds (5%, 10%, 15%, and 20%) at a relatively high intensity (85% 1RM) on PAPE in the lower limb explosive power of sprinters. The principal findings support our hypothesis, demonstrating that the squat protocol employing a 5% VL threshold induces a more effective enhancement of lower limb explosive power compared to the 10%, 15%, and 20% VL conditions. Specifically, at the 4-min recovery mark, the 5% VL protocol elicited significant and superior potentiation in 30-m sprint performance, CMJ height, PPO, and momentum relative to the other VL conditions. Furthermore, the number of squat repetitions completed under this condition was the lowest among the four VL thresholds. These results provide sprint coaches and athletes with evidence-based theoretical and practical guidance for designing lower limb training programs, particularly for optimizing PAPE through precise control of training volume via VL modulation.

### 4.1 Enhancement effects of four velocity loss thresholds on jumping performance

Vertical jump height, PPO, and momentum are considered the most direct indicators of vertical jump performance (Kilduff et al., 2008; Yuan et al., 2023; Liu et al., 2024). The results of this study demonstrated that 4 min after the 5% VL squat intervention, CMJ height, PPO, and momentum were significantly greater than baseline levels, indicating that potentiation outweighed fatigue. These findings are consistent with previous studies (Rojas-Jaramillo et al., 2024; Sánchez-Valdepeñas et al., 2024). Additionally, performance exhibited a progressive decline as VL increased, indicating that greater VL aggravated fatigue and thereby attenuated the potentiation effect. González-Badillo et al. (2017) reported a strong correlation (r^2^ = 0.83) between VL and the percentage of maximum repetitions completed. Weakley et al. (2020) further confirmed that, under identical loads, RPE and blood lactate concentrations increased as VL rose, while immediate jump height declined, supporting the notion that higher VL amplifies fatigue effects.

Muscle contraction is known to simultaneously generate both potentiation and fatigue, with the net manifestation of PAPE dependent on potentiation surpassing fatigue (Brown and Loeb, 1998). Hamada et al. (2003) argued that excessive neural activation may result in intramuscular fatigue predominating, thereby diminishing potentiation. Therefore, selecting an appropriate load is crucial for eliciting PAPE while minimizing fatigue.

The findings of this study provide direct evidence for the balance between fatigue and potentiation regulated by VL in sprint-specific training. The 5% VL protocol successfully induced a significant PAPE effect while avoiding the immediate fatigue-induced decline in performance observed in higher VL conditions, indicating its capacity to minimize fatigue and allow the potentiation effect to dominate motor performance. This study found that near-optimal enhancement occurred as early as the 4-min mark, which differs from the traditional PAPE literature based on high-intensity compound movements, which often suggests an optimal recovery window of 8–12 min. This suggests that the peak potentiation effect may manifest earlier when using a low fatigue inducing protocol.

Placing these findings within the broader context of sprint research further clarifies their significance. The extremely low VL threshold (5%) serves as a precise fatigue-management strategy, and its efficacy is supported by literature affirming that VBT optimizes the management of training volume in contrast to other fatigue-avoidance methods such as post-activation of antagonist muscles (González-Badillo et al., 2017). Concurrently, the shorter ideal recovery window (4–8 min) aligns more closely with findings from studies focusing on sprint-specific activation strategies (Krzysztofik et al., 2022; Stastny et al., 2024). Moreover, the minimal fatigue induced by the 5% VL protocol may help preserve optimal muscle-tendon stiffness, a critical factor for efficient force transmission and elastic energy utilization in jumping, which could be compromised under higher fatigue levels (Pisz et al., 2023). Therefore, the squat protocol employing a 5% VL threshold offers sprinters an efficient, quantifiable, and reliable strategy for potentiating explosive power, characterized by both time efficiency and consistent effectiveness.

### 4.2 Potentiation effects of different velocity loss thresholds on sprint performance

The 30-m sprint results revealed potentiation effects at 4 and 8 min post-intervention following the 5% VL squat protocol, and at 4 min following the 10% VL protocol. This pattern closely mirrored the trends observed in CMJ performance. The emergence of PAPE at 4–8 min of recovery under the 5% VL condition is generally consistent with earlier findings (Gouvêa et al., 2013; Seitz and Haff, 2016; Wilson et al., 2013).

It should be noted, however, that a significant portion of the existing literature framing this discussion is based on traditional PAPE research, often employing fixed-load or repetition-based protocols without precise control over movement velocity during the conditioning activity itself (Xie et al., 2022; Liu et al., 2024). In contrast, the present study advances this discourse by utilizing VL as a direct, real-time metric to regulate the quality of the concentric phase and, by extension, manage the metabolic and mechanical stress of each repetition. This velocity-based approach provides a more nuanced lens to interpret our findings, shifting the focus from solely the temporal characteristics of recovery to the qualitative control of the stimulus that precedes it.

With respect to temporal characteristics, sprint performance improvements occurred between 4 and 8 min post-intervention, corresponding to the time window for enhanced CMJ performance. This temporal alignment suggests that PAPE in sprinting and jumping may arise from similar mechanisms, such as augmented neural drive, elevated muscle temperature, and improved utilization of elastic energy. Immediately following the intervention, fatigue predominates and suppresses performance. However, as recovery progresses, potentiation becomes more influential, with performance peaking within an optimal window before gradually dissipating (Kilduff et al., 2007).

Compared with body weight-based interventions (e.g., sprinting, jumping, plyometric training), which induce minimal fatigue and rapid performance improvements, heavier external loads typically extend the recovery period required for potentiation to outweigh fatigue. Tobin and Delahunt (2014), Sharma et al. (2018) noted that submaximal intensity warm-ups prior to conditioning activity are more likely to produce robust PAPE effects, whereas maximal or exhaustive protocols generate more variable outcomes. Most studies therefore recommend submaximal warm-ups to maximize subsequent potentiation.

Meta-analyses have examined the optimal recovery interval for PAPE (Tillin and Bishop, 2009; Gouvêa et al., 2013; Seitz and Haff, 2016; Dobbs et al., 2019; Wilson et al., 2013). Wilson et al. (2013) proposed 7–10 min (ES = 0.70). Despite methodological differences, most studies agree that the optimal recovery window lies between 3 and 10 min. However, the manifestation of PAPE is highly individualized (Tillin and Bishop, 2009), with each athlete’s optimal interval influenced by factors such as muscle fiber type and strength level. A respondent analysis of our data revealed distinct subgroups that benefited differentially from the 5% velocity loss threshold protocol. This strengthens our conclusion that while the 5% VL condition was optimal on average, individual response variability must be considered to achieve precise programming. In practical application, sprint coaches must identify the precise recovery interval for individual athletes. The finding that a 5% VL protocol is optimal is specific to the present study’s design (2 sets at 85% 1RM with 1-min interest rest). Considering feasibility in real-world training, adopting a 5% VL protocol combined with a 4–8 min recovery interval appears optimal for eliciting PAPE and enhancing sprint performance.

### 4.3 Limitations

Although the present study has clinical relevance, several limitations merit attention. First, the research did not include female sprinters in the activation intervention and analysis. Subsequent studies should conduct in-depth investigations focusing on female athletes. Second, the current study only examined the PAPE effects under relatively high intensity and moderate-to-low velocity loss conditions. Future research could compare low versus moderate-to-high velocity loss conditions to explore the mechanisms of PAPE from multiple perspectives and consider systematically manipulating both velocity loss thresholds and inter-set rest periods (e.g., 1 min vs. 3–5 min) to distinguish between fatigue management effects and the inherent potentiating stimulus of the velocity-based protocol itself. This would help clarify whether the observed outcomes are primarily driven by volume modulated through velocity loss or by the inter-set recovery time. Third, the absence of a control group (e.g., a passive rest condition) limits our ability to definitively attribute the performance changes solely to the PAPE effect elicited by the squat protocols, as opposed to the effects of warm-up or measurement familiarization. Fourth, the reliance on performance-based outcomes without concomitant physiological measurements, such as surface electromyography (sEMG) for neuromuscular activity or muscle temperature, constrains a deeper mechanistic understanding of the observed PAPE responses under different VL thresholds.

## 5 Conclusion

This study demonstrated that VBT protocols designed to induce PAPE can significantly enhance sprint performance. For practical application in training or competition, and within the specific context of the protocol applied here (2 sets at 85% 1RM with 1-min interest rest), it is recommended that practitioners employ a 5% VL load intensity with a recovery interval of 4–8 min following the conditioning activity to optimize performance outcomes. Establishing this dose-response relationship offers a robust framework for regulating neuromuscular activation thresholds, optimizing the temporal management of acute enhancement effects, and ultimately maximizing competitive performance in sprinters.

## Data Availability

The raw data supporting the conclusions of this article will be made available by the authors, without undue reservation.
